# Publications on community psychiatry

**DOI:** 10.4103/0019-5545.69248

**Published:** 2010-01

**Authors:** R. Thara, Sushma Rameshkumar, C. Greeshma Mohan

**Affiliations:** Schizophrenia Research Foundation (SCARF), Chennai, India

**Keywords:** Community psychiatry, programs, publications

## Abstract

Care and treatment outside the setting of mental hospitals have been termed community psychiatry. This paper, based largely on publications on this subject in the IJP, discusses work on development of mental health services outside the hospitals, National and District Mental Health Programs, some accounts of Indian families, alternative modes of treatment in communities and a few miscellaneous issues. Very few papers are data driven and most of them are descriptive and opinionated.

## IJP-PUBLICATIONS- COMMUNITY MENTAL HEALTH

Before I proceed to define and discuss research in Community Psychiatry in India, I wish to state that this review, based only on publications in the IJP, is by no means comprehensive. It does not include the large body of Indian work which has been published in other journals such as the British Journal of Psychiatry, World Psychiatry, International J of Social Psychiatry, Int. Review of Psychiatry and many others. This also does not include abstracts of papers presented at the various conferences of the Indian Psychiatric Society, since they are not truly peer reviewed publications. Since not much of actual research has been done on this subject and most of the papers are descriptive accounts or view points, I prefer to refer to it as publication rather than research.

## INTRODUCTION

The word community means different things to different people, but is generally used to denote a particular geographical and administrative area, a relatively wel-integrated neighbourhood and locality. It also alludes to areas outside big hospitals.

Community Psychiatry (CP) has been defined in many ways. While it originates from certain historical background of “deinstitutionalization” in western countries, it generally has denoted the development of services in many of the developing countries. Many developing countries including India did not have an adequate number of institutions to care for the mentally ill. Most care in fact took place in the community and family. This was with or without the involvement of mental health services depending on their availability.

This is why the term community psychiatry in India alludes to establishment of new services/programmes in the community rather than the process of de-institutionalization.

Szmukler and Thornicroft[1] define community psychiatry as follows:

“Community psychiatry comprises the principles and practices needed to provide mental health services for a local population by (i) establishing population-based needs for treatment and care; (ii) providing a service system linking a wide range of resources of adequate capacity, operating in accessible locations and (iii) delivering evidence based treatments to people with mental disorders

These ‘principles’ of community psychiatry, proposed by Caplan and Caplan,[2] have also proved useful and valid to varying degree in defining the subject. These principles include:

1. Responsibility to a population, usually a catchment area defined geographically
2. Treatment close to the patient’s home
3. Multi-disciplinary team approach
4. Continuity of care
5. Consumer participation
6. Comprehensive services

## INDIA

Over the last three to four decades, community psychiatry in India has made substantial advances. When mental hospitals were found to be inadequate, a number of initiatives including the National Mental Health Programme were started as community based activities. Now, it appears that about 20% of the country has had the benefit of integration of mental health with general medical services in about 100 districts all over the country. There are also initiatives by non-governmental organizations and private institutions.

Given this background and keeping in mind the inevitable overlap with other chapters in this volume, the issue covered will be the following (not necessarily in that order):

1. Development of mental health services
2. National Mental Health Programme (NMHP) and the District Mental Health Programme (DMHP)
3. Primary care psychiatry
4. Families
5. Other miscellaneous topics

### Community MH Programmes

The late Dr. R.L. Kapur[3] who has written extensively on community mental health, began the story of community psychiatry with Dr. Vidya Sagar who, as early as in the 1950s, involved family members of patients admitted into Amritsar Mental Hospital. This experiment not only reaped rich benefits but also initiated a major movement of involving families in the care process. Now family wards are located in several institutions like NIMHANS, CMC, Vellore and the IMH, Chennai.

There have been some long standing, well known community-based projects in India. One of them is the Raipur Rani experiment. Wig *et al*. in 1981[4] described in great detail the entire study of over 60,000 persons in Haryana, the methodology, the tools used and the means in which the community was involved in all activities. The same authors have also written on the organization of mental health services through primary/peripheral care centres based on the Raipur Rani experience.

The other well known community study has been at Sakalwara in Karnataka. Chandrasekar *et al*.[5] describe the three-year experience in a paper published in 1981 and the lessons that can be learnt from this. From the same centre, Mohan Isaac *et al*. describe the development and evaluation of a training programme to effect mental health care delivery through a PHC in rural Karnataka.[6]

The other large studies have been the one at Barwani by Chatterjee *et al*.[7] and at Thiruporur by SCARF.[8] Publications on these can be found in other journals.

## MENTAL HOSPITALS

In their interesting article on Agra mental hospital, Sudhir Kumar and Rakesh Kumar (2008) describes the growth of what was called lunatic asylums in India during the British rule. The institution went through many phases including what they term the golden period when K.C. Dube was at the helm of affairs-characterized by a lot of international research including the well known WHO study the IPSS. Now, community care is well developed in Agra and the institute itself has a department of research and rehabilitation.[9]

O. Somasundaram has in an interesting publication named “Asylums and authors” described the several historical figures associated with mental asylums.[10]

## NATIONAL MENTAL HEALTH PROGRAMMES

There have been a lot of descriptive papers on NMHP and DMHP. Unfortunately, very few have been data-based and few published in the IJP; a mid-term appraisal by Murthy makes interesting reading.[11]

Three other publications from NIMHANS deal with other issues like management of priority mental disorders in a community, evaluation of a pilot training programme and a follow-up of rural mentally ill.[12–14]

## MENTAL HEALTH SERVICES

Desai[15] traced the development of mental health services in India and made some comparisons. He argued that a “truly comprehensive and meaningful conceptualization of community mental health can also include many of these aspects, and that public mental health is no more than community mental health”. He also opined that attention should be paid to social issues such as poverty, homelessness, violence, urbanization homeless populations, refugees, disaster affected populations, as also the health issues of the affluent classes “.

The next phase of community-based services started with the beginning of psychiatric units in general hospitals (GHPUs)., Wig in his 1978 publication mentioned that the first such unit was set up in 1933 at the R.G.Kar Medical College at Kolkata followed by many others in the 60s. There seem to be close to 300 GHPUs now.[16]

## PRIMARY CARE PSYCHIATRY

It has been suggested that in many developing countries, primary care psychiatry can effectively replace the term community psychiatry. In developing countries, where the number of specialist mental health professionals is very small in comparison to the actual demand, the provision of mental health services would remain a dream unless psychiatry was firmly rooted in primary health care.

Training programmes form a critical part of primary care psychiatry involving as it does training of several types of professionals such as PHC doctors and nurses, and Community level Workers. In 1989, Shamsundar *et al*.[17] described the training programme of GPs based on a ICMR study. This was a multi site study initiated by ICMR “Training Programme in Psychiatry for non psychiatrist primary care doctors” and was held in 1982-83. Manuals were developed and an evaluation of the training programme done.

Murthy and colleagues[18] describe the use of case vignettes for the assessment of GPs during their training. In 1985, Gautam[19] discussed the development and evaluation of training programmes for primary care in India. The other publications on training have been by Devi (short term training), and Nagarajiah *et al*. on evaluation of such training.[20,21]

Inspired by eye camps, psychiatric camps were organized in places where the distance to the nearest psychiatric facility was long. The village leaders were also involved in the therapeutic process and in stigma reduction efforts.[22]

Community mental health also includes school mental health. This was pioneered at NIMHANS by Malavika Kapur. School Mental Health is an important element of community psychiatry. The programme at NIMHANS developed manuals, trained school teachers to diagnose children with emotional problems and to counsel them. It was evaluated with satisfactory results.[23]

## ALTERNATIVE MEDICAL PRACTICES AND NON MEDICAL HEALING

It is widely known that even today religious healing takes place in select temples and durghas. The mode of healing varies from place to place. The entire country was put to shame by the Erwadi tragedy which saw 26 patients chained in a hut close to a religious healing site in Tamil Nadu who were burnt to death by an accidental fire which broke out one night.

The Erwadi tragedy and its implications have been well written by Trivedi in an editorial[24] and James Anthony[25] in a long letter to the editor in 2002. Both while lamenting the inhuman tragedy have urged the mental health community and policy makers to have a relook at the framework of mental health services in general and community psychiatry in particular.

Somasundaram has given a very lucid account of religious treatment of the mentally ill in several Hindu temples and durghas in Tamilnadu.[26] Joel *et al*. conducted in CMC, Vellore a bio medical educational intervention to change explanatory models of psychosis among CLWs in south India.[26]

## FAMILIES

Families in general and those of the mentally ill have been a subject of some research. There have been some general accounts of families in India. Ramanujam[27] dealt with the psychology and sociology of families. As early as 1967, Rose Chacko underscored the importance of the Indian family in the process of psychosocial rehabilitation.[28]

In 1967, Sethi BB conducted a community survey on 300 urban families in Lucknow studying social and demographic features of families.[29] The same author also discussed in his 1978 publication the need to understand the social processes in India to care better for the mentally ill.[30] In 1981, he wrote an editorial on the subject of family planning and its implications for mental health.[31]

## MISCELLANEOUS

Medical practices among an Indian tribe have been described by Vinod Kumar Singh in 1972.[32] An interesting epidemiological study of a Himalayan tribe by Ghosh *et al*. found psychiatric morbidity in 50% of the tribe with more women being affected.[33] B oral *et al*. studied different treatment methods used by the mentally ill and their social acceptance.[34]

Srinivasan and Suresh[35] conducted a study on the prevalence of non psychotic mental morbidity in a primary care setting and have identified variables such as female sex, unskilled labour, younger age etc to be more associated with this. In 2004, Roy Abraham elucidated the mental health issues in the south Asian region.[36] In the same year, Srinivasa Murthy detailed the challenges and resources in the utilization of human resources in mental health care.[37]

In the editorial on the impact of rapid urbanization on mental health, Trivedi *et al*.[38] opined that South-Asian countries by virtue of their developing economies and poverty level are particularly vulnerable and tend to have a higher burden of diseases with an already compromised primary health care delivery system. The range of disorders and deviancies associated with urbanization is enormous and includes psychoses, depression, sociopathy, substance abuse, alcoholism, crime, delinquency, vandalism, family disintegration, and alienation.

Seasonal variation was reported by Singh *et al*.[39] Their study showed a significant relationship between utilization of psychiatric patients, especially with mood disorders and neurotic, stress related and somatoform disorders with season (summer and autumn respectively).

Some innovations in mental health have also been described.[40] One of them is the establishment of ambulatory services for the mentally ill.

For the first time, Karnataka State Mental Health Authority (KSMHA), in coordination with Government of Karnataka, Rotary Club, Bangalore West, and ACMI, a non-governmental organization (NGO) began such a service in the city of Bangalore. The request for such services had come from NGOs formed by families of the mentally ill. Guidelines were framed and approved by the KSMHA. Funding for this project has come from NGOs and the KSMHA. This has been functioning from October 2008 onwards. It appears that this is a model of community care which can be replicated in other parts of India as well.

## NON-GOVERNMENTAL ORGANIZATIONS

In the last two decades, NGOs working on mental health have played a major role in filling many gaps in community mental health. These have ranged from rehabilitation and after care of severely disabled persons with psychoses, to children, substance abuse, elderly and suicide. A book by Patel and Thara[41] provides a comprehensive account of the role of NGOs in India.

## CONCLUSION

It can be said that community psychiatry has been evolving slowly, but surely, over the last few decades. It has largely been marked by anecdotal reports, program descriptions and individual descriptions. The NMHP and DMHP have been erratic in their functioning and have not generated much evaluable data. There is no central, unified policy on community mental health in India. Unless this happens, it is likely that publications on community psychiatry will continue to be descriptive and anecdotal.
